# Utility of *In Vivo* Transcription Profiling for Identifying *Pseudomonas aeruginosa* Genes Needed for Gastrointestinal Colonization and Dissemination

**DOI:** 10.1371/journal.pone.0015131

**Published:** 2010-12-10

**Authors:** Andrew Y. Koh, Per J. Mikkelsen, Roger S. Smith, Kathleen T. Coggshall, Akinobu Kamei, Michael Givskov, Stephen Lory, Gerald B. Pier

**Affiliations:** 1 Department of Pediatrics, University of Texas Southwestern Medical Center, Dallas, Texas, United States of America; 2 Department of Hematology/Oncology, University of Texas Southwestern Medical Center, Dallas, Texas, United States of America; 3 Department of Infectious Diseases, University of Texas Southwestern Medical Center, Dallas, Texas, United States of America; 4 Department of Microbiology, University of Texas Southwestern Medical Center, Dallas, Texas, United States of America; 5 Department of International Health, Immunology and Microbiology, University of Copenhagen, Copenhagen, Denmark; 6 The Broad Institute of MIT and Harvard, Cambridge, Massachusetts, United States of America; 7 University of California San Francisco School of Medicine, San Francisco, California, United States of America; 8 Channing Laboratory, Department of Medicine, Brigham and Women's Hospital, Harvard Medical School, Boston, Massachusetts, United States of America; 9 Department of Microbiology and Molecular Genetics, Harvard Medical School, Boston, Massachusetts, United States of America; Institut de Pharmacologie et de Biologie Structurale, France

## Abstract

Microarray analysis of *Pseudomonas aeruginosa* mRNA transcripts expressed *in vivo* during animal infection has not been previously used to investigate potential virulence factors needed in this setting. We compared mRNA expression in bacterial cells recovered from the gastrointestinal (GI) tracts of *P. aeruginosa*-colonized mice to that of *P. aeruginosa* in the drinking water used to colonize the mice. Genes associated with biofilm formation and type III secretion (T3SS) had markedly increased expression in the GI tract. A non-redundant transposon library in *P. aeruginosa* strain PA14 was used to test mutants in genes identified as having increased transcription during *in vivo* colonization. All of the Tn-library mutants in biofilm-associated genes had an attenuated ability to form biofilms *in vitro*, but there were no significant differences in GI colonization and dissemination between these mutants and WT *P. aeruginosa* PA14. To evaluate T3SS factors, we tested GI colonization and neutropenia-induced dissemination of both deletional (PAO1 and PAK) and insertional (PA14) mutants in four genes in the *P. aeruginosa* T3SS, *exoS* or *exoU*, *exoT*, and *popB*. There were no significant differences in GI colonization among these mutant strains and their WT counterparts, whereas rates of survival following dissemination were significantly decreased in mice infected by the T3SS mutant strains. However, there was a variable, strain-dependent effect on overall survival between parental and T3SS mutants. Thus, increased transcription of genes during *in vivo* murine GI colonization is not predictive of an essential role for the gene product in either colonization or overall survival following induction of neutropenia.

## Introduction

Modern molecular tools allow for analysis of levels of mRNA in bacterial cells living in different environments and many such studies have been applied to *Pseudomonas aeruginosa* [1], [2], [3], [4], [5]. However, apart from one study analyzing mRNA expression in *P. aeruginosa* present in the sputum of a single cystic fibrosis patient [6], no other studies have analyzed *P. aeruginosa* gene expression during *in vivo* infection. Part of the problem lies with recovering sufficient mRNA from bacterial cells directly isolated from infected tissues for microarray analysis. In a previously described model of murine GI colonization and dissemination following induction of neutropenia [7] we found that high levels of *P. aeruginosa* could be recovered from the mouse cecum, potentially identifying a source of *in vivo* bacterial mRNA sufficient for microarray analysis. This model mimics the morbidity and mortality of immunocompromised hosts such as patients with leukemia, severe burn wounds or recipients of organ transplants [8]. In many patients at-risk for *P. aeruginosa* infection (i.e. surgical patients, cancer patients receiving chemotherapy) the gastrointestinal (GI) tract is believed to be the main tissue initially colonized by this organism, often times allowing for translocation to extra-gastrointestinal sites and, in the worst cases, development of life-threatening sepsis [9], [10]. In this patient group, *P. aeruginosa* has the highest case-fatality rate among all gram-negative pathogens [11]. The mere presence of *P. aeruginosa* in the GI tract of critically-ill surgical patients is associated with a 70% mortality rate, a three-fold increase over physiologically matched critically-ill patients not infected with *P. aeruginosa* [12].

Here we report that enough *in vivo*-expressed *P. aeruginosa* mRNA can be recovered from microbial cells in the cecum of colonized mice and determined that genes involved in two major bacterial phenotypes thought to be important in virulence, production of biofilms and elaboration of type III secretion system (T3SS) effectors, had consistent increases in mRNA levels when compared with expression levels in the bacteria living in the water used to colonize the mice. Using *P. aeruginosa* strains from both a non-redundant transposon insert library in strain PA14 [13] as well as deletional mutants in strains PAK and PA01, we confirmed that the genes with increased transcription that were involved biofilm production were deficient in this phenotype *in vitro*, but were not compromised in either their ability to colonize the GI tract *in vivo* or disseminate following induction of neutropenia. Mutants in T3SS effector genes were also not deficient for GI colonization. Mice infected with these mutants had an increase in time of survival after induction of neutropenia but variable, strain-dependent effects on overall mouse survival with different T3SS mutants. Our findings validate a system for measuring gene expression of *P. aeruginosa* during an important phase of the pathogenesis of human disease, but also indicate that increased mRNA expression is not necessarily predictive of a phenotype needed by this organism to induce or maintain *in vivo* infection or cause WT levels of pathology.

## Results

### Transcription Profiling of *P. aeruginosa* in the Murine GI Tract

**Figure 1:**
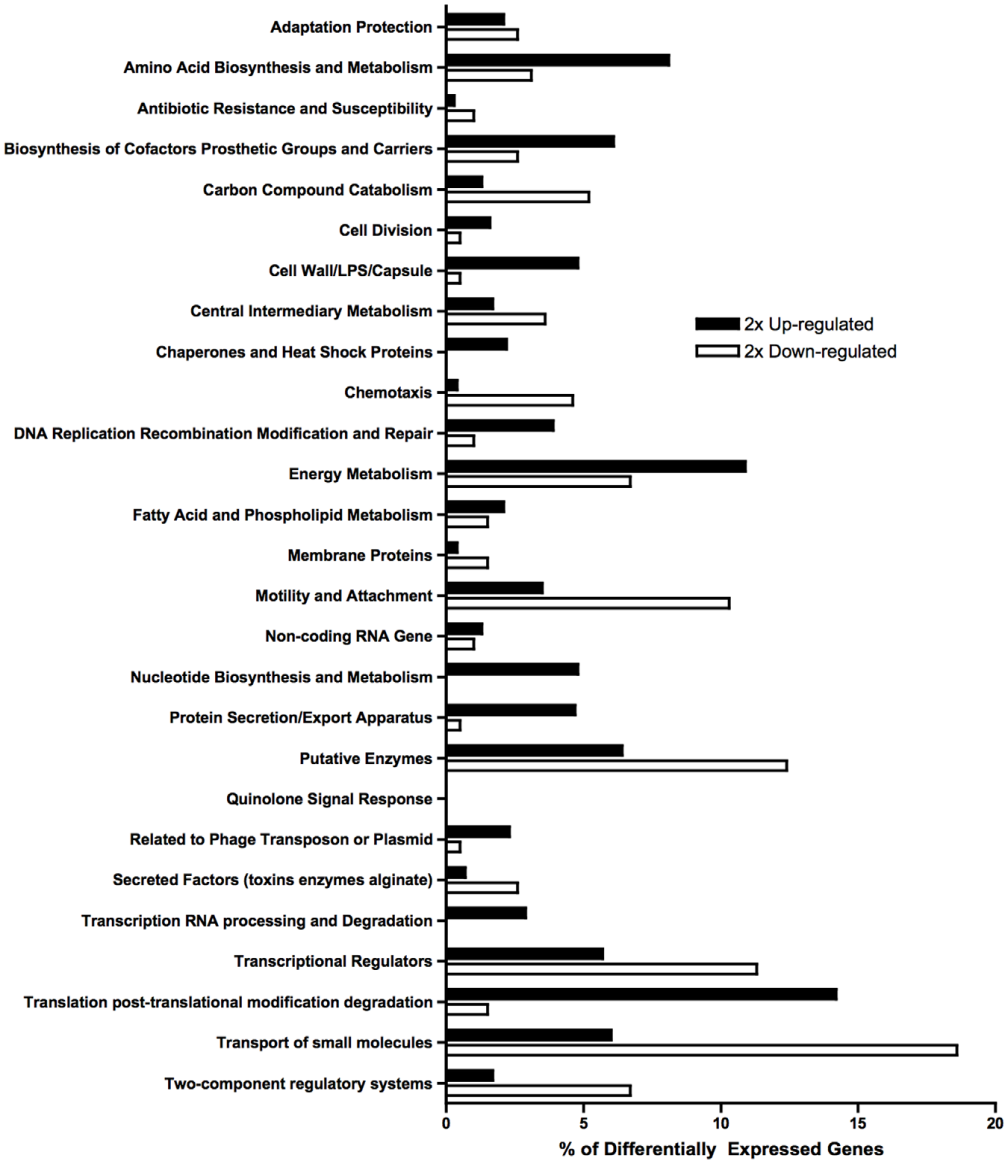
Functional classification of *P. aeruginosa* (PAK strain) genes differentially expressed in murine gastrointestinal colonization. The functional classes are according to the *Pseudomonas* Community Annotation Project (www.pseudomonas.com). The percentage of differentially expressed genes is calculated as the number of genes in each class divided by the total number of differentially 2× up-regulated or 2× down-regulated genes. Note that many genes have more than one functional annotation. LPS, lipopolysaccharide.

We initially identified *P. aeruginosa* genes that were differentially transcribed in the GI tract of eight colonized C3H mice in comparison to genes expressed in sterile water containing 1500 U penicillin G/mL. For both the basal state and colonization states, three separate biologic replicates were performed. We chose to use *P. aeruginosa* recovered from the drinking water as the baseline condition since water contaminated with *P.aeruginosa* is often cited as the source for both nosocomial and community-acquired *P. aeruginosa* infections. Within the hospital, *P. aeruginosa* is most often isolated from water sources such as sinks, drains, toilets, and showers [14] and has been identified by molecular techniques (e.g. genotyping) to be the causative pathogen in documented *P. aeruginosa* infections [15]. Furthermore, community-acquired *P. aeruginosa* infections are also often associated with exposure to contaminated water sources (i.e. swimming pools, hot tubs) [16]. Given the epidemiology of *P. aeruginosa* acquisition and the fact that our murine model was designed with the aim of recapitulating the pathogenesis of *P. aeruginosa* infection in a human host, we felt that using bacteria recovered from the drinking water was the best representative of a baseline condition. Although the *P. aeruginosa* arrays were developed using the PAO1 genome [17], previous studies have shown that the strain PAK transcriptome of the genes conserved between these two strains can be reliably measured with *P. aeruginosa* Genome Arrays (of the 5886 *P. aeruginosa* probe sets included on the microarray, 5678 were detected using PAK DNA) [18]. An average of the microarray hybridization intensity data (3 microarrays) for both baseline (drinking water) and colonization state (cecums) was generated and compared (Gene Expression Omnibus Accession Number GSE22665). Statistical analysis of the derived transcriptomes indicated that 1089 genes (including 321 hypothetical and unclassified genes) showed a two-fold increase in expression from the baseline to the GI colonization state, and 423 genes (including 229 hypothetical and unclassified genes) showed a two-fold decrease in expression from baseline (Figure 1).

We found that the transcript levels of genes functionally classified as being involved in Cell Wall/LPS/Capsule were generally increased by two-fold or more over expression when the bacteria are in water: 34 of 86 genes (40%) involved in Cell Wall/LPS/Capsule function were expressed at a higher level in the GI tract versus 2 of 86 (2%) genes expressed at a <2-fold lower level. In contrast, relatively few genes involved in membrane protein synthesis were changed (3 of 43 increased versus 7 of 43 decreased), and genes involved in motility and attachment were roughly evenly increased or decreased (18 of 67 genes had increased mRNA levels while 20 of 67 genes had decreased mRNA levels). Of the 16 biofilm genes that we chose to study, 11 of 16 genes (69%) were expressed at >2-fold level in the GI tract compared to expression levels of bacteria in water. Most interesting, however, was that 98% of the genes (39 of 40) involved in the Type III Secretion System had increased expression and none had decreased expression (Table 1). Of the 5 T3SS genes that we studied, all of the genes (with the exception of *exoU,* since *exoU* is absent in strain PAK) showed a seven-fold or higher increase in transcript levels (Table 1).

**Table 1:** Type III Secretion Gene Expression in *P. aeruginosa* murine GI colonization.

Gene Name	Function	Fold Activation Mean (SEM)
exoS (PA3841)	exoenzyme S	25.5 (3.4)
exoT (PA0044)	exoenzyme T	18.9 (0.9)
exoY (PA2191)	adenylate cyclase ExoY	7.2 (0.1)
exsA (PA1713)	transcriptional regulator ExsA	10.0 (0.6)
exsB (PA1712)	exoenzyme S synthesis protein B	3.3 (0.6)
exsC (PA1710)	exoenzyme S synthesis protein C precursor	3.1 (0.2)
exsD (PA1714)	hypothetical protein	7.1 (1.2)
orf1 (PA3842)	probable chaperone	14.4 (1.7)
pcr1 (PA1699)	conserved hypothetical protein in type III secretion	19.6 (5.1)
pcr2 (PA1700)	conserved hypothetical protein in type III secretion	11.0 (1.8)
pcr3 (PA1701)	conserved hypothetical protein in type III secretion	10.7 (1.6)
pcr4 (PA1702)	conserved hypothetical protein in type III secretion	13.1 (1.8)
pcrD (PA1703)	type III secretory apparatus protein PcrD	8.8 (0.8)
pcrG (PA1705)	regulator in type III secretion	3.9 (0.1)
pcrH (PA1707)	regulatory protein PcrH	9.5 (0.7)
pcrR (PA1704)	transcriptional regulator protein PcrR	2.9 (0.3)
pcrV (PA1706)	type III secretion protein PcrV	8.5 (1.2)
PopB (PA1708)	translocator protein PopB	8.9 (1.1)
PopD (PA1709)	Translocator outer membrane protein PopD precursor	10.1 (1.3)
PopN (PA1698)	Type III secretion outer membrane protein PopN	12.7 (2.7)
pscB (PA1715)	type III export apparatus protein	16.5 (4.0)
pscC (PA1716)	Type III secretion outer membrane protein PscC precursor	10.1 (1.0)
pscD (PA1717)	type III export protein PscD	12.8 (2.0)
pscE (PA1718)	type III export protein PscE	10.4 (2.5)
pscF (PA1719)	type III export protein PscF	16.4 (3.8)
pscG (PA1720)	type III export protein PscG	10.9 (1.4)
pscH (PA1721)	type III export protein PscH	10.1 (2.0)
pscI (PA1722)	type III export protein PscI	7.5 (2.6)
pscJ (PA1723)	type III export protein PscJ	8.1 (1.8)
pscK (PA1724)	type III export protein PscK	6.3 (0.9)
pscL (PA1725)	type III export protein PscL	5.2 (1.5)
pscN (PA1697)	ATP synthase in type III secretion system	9.1 (0.4)
pscO (PA1696)	translocation protein in type III secretion	15.5 (1.4)
pscP (PA1695)	translocation protein in type III secretion	58.8 (2.9)
pscQ (PA1694)	translocation protein in type III secretion	9.9 (2.3)
pscR (PA1693)	translocation protein in type III secretion	5.7 (0.7)
pscS (PA1692)	probable translocation protein in type III	4.0 (0.1)
pscT (PA1691)	translocation protein in type III secretion	2.8 (0.7)
PA1711	hypothetical protein	3.6 (0.3)

### Selection of *P. aeruginosa* PA14 strains with insertions in genes needed for biofilm formation

Using the data generated from several studies [19], [20], [21], [22], we identified genes that have been shown to be important for all stages of *in vitro* biofilm formation: *fleR, flgK,fliP, sadB, crc, pilB, pilC, pilY1, PA3782, lasI, rhlA, and rpoN.* Next, we cross-referenced the murine GI colonization transcriptome data that we generated with three published transcription profiling studies related to *P. aeruginosa* biofilm formation *in vitro* [2], [4], [5]. Two genes encoding hypothetical proteins (*PA0713* and *PA0952*) were significantly increased in 2 or more datasets (Table 2), and two additional genes encoding hypothetical proteins were found to be important in biofilm formation using a signature-tagged mutagenesis analysis of factors needed for virulence in a rat *P. aeruginosa* pulmonary lung infection [23] model: *PA0141* (25 fold increase) and *PA1009* (4.8 fold increase). The transcriptome results generated from murine GI colonization for genes known to be involved in biofilm formation *in vitro* and for the genes encoding hypothetical proteins are provided in Table 2.

**Table 2:** Biofilm genes differentially expressed in *P. aeruginosa* murine GI colonization.

Gene Name	Function	Fold activation (mean, SEM)			
		This study	Hentzer *et al.*	Waite *et al.* 8 hours*	Waite *et al.* 48 hours*
*PA0713*	Hypothetical	24.8 (2.0)	2.8	3.4	3.7
*PA0952*	Hypothetical	15.6 (4.7)	4.2	4.4	2.0
*PA0141*	Hypothetical	24.8 (0.9)	3.0	0.1	0.8
*PA1009*	Hypothetical	4.9 (0.1)	--------	1.9	1.0
*crc* (*PA5332*)	Catabolite repression control protein	1.9 (0.1)	------	0.4	0.4
*fleR (PA1099)*	Two-component response regulator	0.3 (0.03)	------	0.7	0.1
*flgK (PA1086)*	Flagellar hook-associated protein 1	3.1 (1.0)	------	0.6	0.2
*fliP (PA1446)*	Flagellar biosynthetic protein	0.3 (0.02)	------	0.6	0.4
*PA3782*	Probable transcriptional regulator	2.1 (0.3)	------	0.5	1.1
*pilB (PA4526)*	Type 4 fimbrial biogenesis protein	2.8 (0.7)	------	4.5	2.3
*pilC (PA4527)*	Type 4 fimbrial biogenesis protein	6.5 (3.7)	--------	3.1	2.1
*pilY1 (PA4554)*	Type 4 fimbrial biogenesis protein	4.5 (0.7)	------	4.1	1.7
*sadB (PA5346)*	Predicted signal transduction protein	2.7 (0.5)	------	0.9	1.3
*lasI (PA1432)*	Autoinducer synthesis protein	0.4 (0.02)	------	1.3	7.0
*rhlA (PA3479)*	rhamnosyltransferase chain A	0.03 (0.01)	------	3.0	1.1
*rpoN (PA4462)*	RNA polymerase sigma-54 factor	2.1 (0.3)	------	0.8	0.5

*Data was obtained from supplementary tables S4-S10 in Waite *et al*. (available at http://www.biomedcentral.com/content/supplementary/1471-2164-7-162-S3.xls) and fold regulation was calculated by dividing LP signal with 8 h or dividing SP signal with 48 h biofilm signal or vice versa if expression was down-regulated.

### Microtiter Biofilm Assay

**Figure 2:**
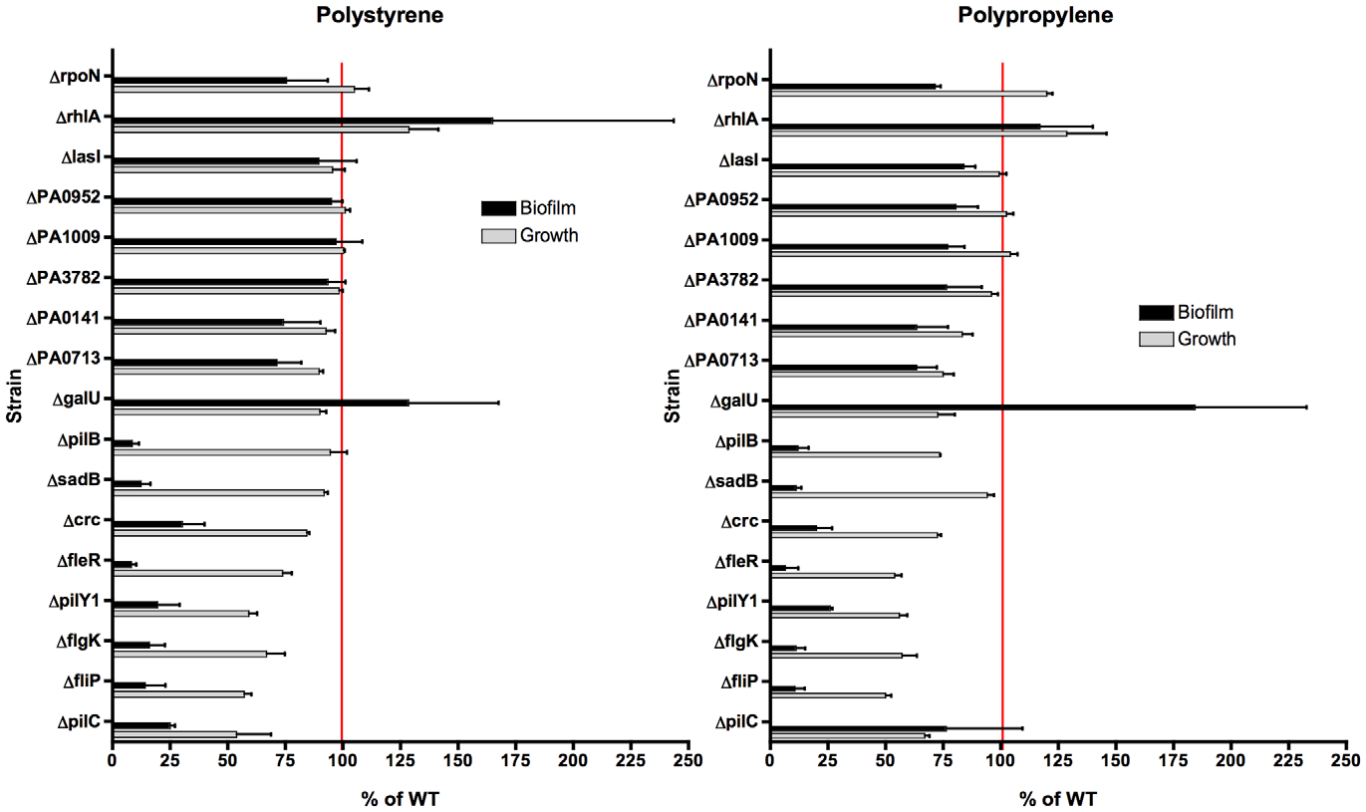
Microtiter Plate Biofilm Assay. *P. aeruginosa* wild-type strain PA14 and the listed mutants (obtained from a non-redundant PA14 transposon-insertion mutant library) were incubated in 96-well microtiter plates with different substrates (polystyrene and polypropylene) for 12–14 hours at 37°C. Growth was measured by spectrophotometry (OD_630_). To assess biofilm formation, microtiter plates were then emptied, washed with sterile 0.9% normal saline, incubated with 1% crystal violet for 15 minutes at RT, then destained with 95% ethanol. The results are the means of three separate biological experiments. Each biological experiment contained 7 technical replicates; the mean was obtained for each biological experiment. The red line indicates biofilm and growth rate for wild-type strain PA14 (100% WT).

**Figure 3:**
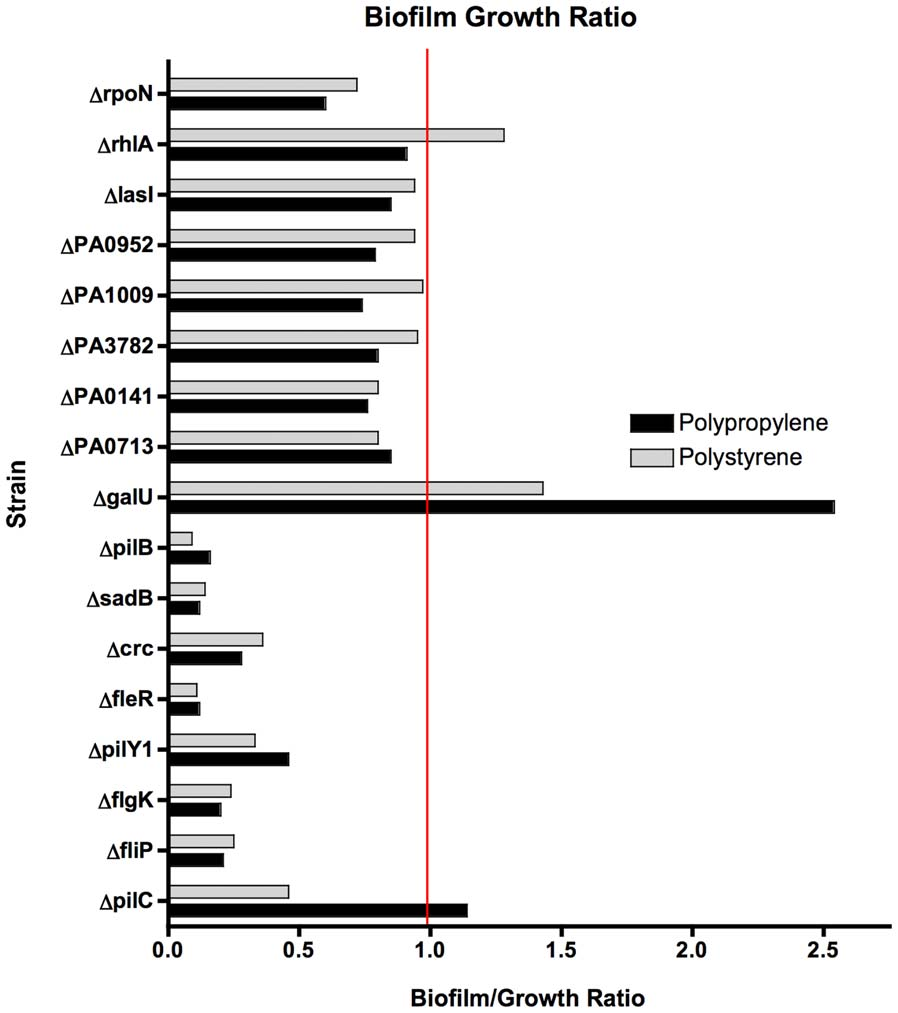
Biofilm/Growth Ratios for *P. aeruginosa* PA14 transposon-insertion mutants listed above. Bars represent the means for three separate biological replicates (each biological replicate is the mean of seven technical replicates). Ratios <1 indicate decreased biofilm formation relative to growth level. By definition WT biofilm/growth ratio is 1, as biofilm and growth levels were used to calculate the ratio are in % of WT. The red line indicates biofilm growth ratio of 1.0.

We then used a non-redundant transposon mutant library in *P. aeruginosa* strain PA14 [13], to retrieve mutant strains with transposon insertions in the candidate genes identified above (Table 2). The disruption of the target genes by the transposon was confirmed by colony PCR. Next, the mutants were tested for biofilm formation *in vitro* using a 96-well microtiter plate assay [20] on two different substrates: polystyrene (PS) and polypropylene (PP). Several strains (i.e., PA14*fliP*, *fleR* and *flgK*) were attenuated in both *in vitro* biofilm formation and growth rates (Figure 2). Assuming a linear relationship between growth and biofilm formation, biofilm to growth ratios were calculated to account for differences in growth (Figure 3). Ratios less than 1 compared to that of the wild-type strain were designated as indicative of decreased biofilm formation. The same PA14 mutants *(*with insertions in *pilB*, *sadB*, *crc*, *fleR*, *pilY1*, *flgK*, *fliP*, and *pilC genes*) that showed significant absolute decreases in biofilm formation also had biofilm:growth ratios less than 1. (Fig. 3). We also analyzed biofilm formation by the *P. aeruginosa* strain PA14*galU^−^* which is unable to synthesize a complete outer core lipopolysaccharide (LPS) [24], as this strain is unable to colonize the antibiotic-treated murine GI tract [7]. Interestingly, the *galU* mutant had a biofilm:growth ratio of 2.5 on PP, making it a better biofilm former than the WT strain PA14.

We also tested biofilm formation under anaerobic conditions (to better approximate conditions found in the human gastrointestinal tract[25]) on both PP and PS substrates. The 8 mutants (*pilB*, *sadB*, *crc*, *fleR*, *pilY1*, *flgK*, *fliP*, and *pilC*) had comparable biofilm formation and biofilm/growth ratios when grown anaerobically as those produced under aerobic growth conditions (data not shown).

### Colonization and Systemic Dissemination from the Murine GI Tract by Biofilm-deficient TN-mutants

**Figure 4:**
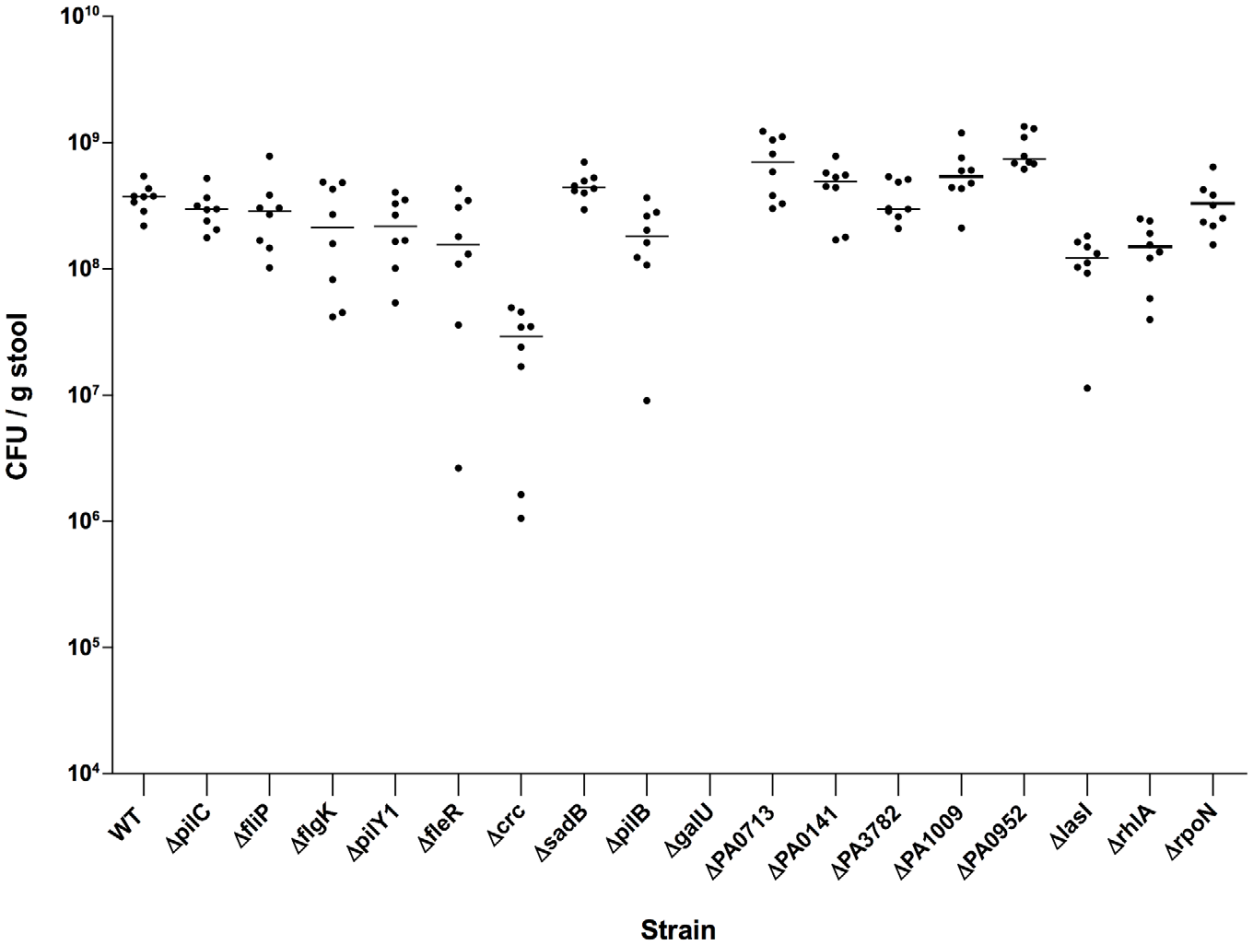
Gastrointestinal Colonization Levels Mice fed *P. aeruginosa* strains PA14 and the above listed PA14 transposon insertion mutants. Points represent result from individual animals (female 6–8 wk-old C3H/HeN mice), and horizontal lines represent the medians. n = 8 for each strain. Colonization levels with strain Δ*crc* were significantly lower (p<0.05 by Kruskal Wallis with Dunn's multiple comparison test) compared with wild-type strain PA14 (WT).

The strains identified as attenuated for biofilm formation *in vitro* were further investigated for colonization and dissemination phenotypes in the murine GI tract colonization and dissemination model [7]. Wild-type *P. aeruginosa* PA14 consistently colonized the mouse GI tract at levels comparable to those previously reported [7] for *P. aeruginosa* strain PAO1: PA14 (n = 8, median = 3.76×10^8^ cfu/g stool, first quartile = 2.84×10^8^, third quartile = 5.24×10^8^) and PAO1 (n = 8, median = 3.52×10^8^ cfu/g stool, first quartile = 2.64×10^8^, third quartile = 3.81×10^8^, Fig. 4). All mutant strains colonized the murine GI tract at comparable levels to WT *P. aeruginosa* PA14, with the exception of the PA14*crc-* mutant and the negative control strain, PA14*galU-*, which does not colonize at detectable levels (Fig. 4). The PA14*crc-* mutant did colonize the GI tract but at significantly lower levels (slightly more than one-log) compared to WT PA14: *crc* (n = 8, median = 2.93×10^7^ cfu/g stool, first quartile = 1.31×10^7^, third quartile = 3.77×10^7^; PA14 (n = 8, median = 3.15×10^8^ cfu/g stool, first quartile = 3.02×10^8^, third quartile = 3.60×10^8^, p = 0.0002 by Mann Whitney test compared to PA14Δ*crc*, Fig. 4). Confirmation of maintenance of the mutant genotype was obtained by performing PCR on colonies recovered from murine feces.

In additional experiments, after establishing and confirming *P. aeruginosa* GI colonization, mice were made neutropenic by administering a single 200 µg injection of monoclonal antibody RB6-8C5 [7], [26]. One-hundred percent of mice colonized with WT PA14 (n = 8) were euthanized when moribund or died in between observation periods. Seventy-five to 100% of mice colonized with all 16 of the PA14 strains with transposon insertions (n = 8 for each mutant strain) in the genes needed for full biofilm formation also became moribund and were euthanized or died in between observation periods. Thus, there were no significant differences in mortality in mice colonized with either WT PA14 or Tn-mutant strains unable to produce biofilms in vitro (Table 3).

**Table 3:** Survival of neutropenic mice after administration of 0.2 mg RB6-8C5 MAb i.p.

Colonizing Strain of *P. aeruginosa*	No. of survivors/No. challenged mice
Wildtype PA14	0/8
PA14*crc-*	0/8
PA14*fleR-*	0/8
PA14*flgK-*	1/8
PA14*fliP-*	0/8
PA14*pilB-*	0/8
PA14*pilC-*	2/8
PA14*pilY1-*	1/8
PA14*sadB-*	0/8
PA14*PA0141-*	2/8
PA14*PA0713-*	0/8
PA14*PA0952-*	1/8
PA14*PA1009-*	0/8
PA14*PA3782-*	2/8
PA14*lasI-*	0/8
PA14*rhlA-*	0/8
PA14*rpoN-*	1/8

**Figure 5:**
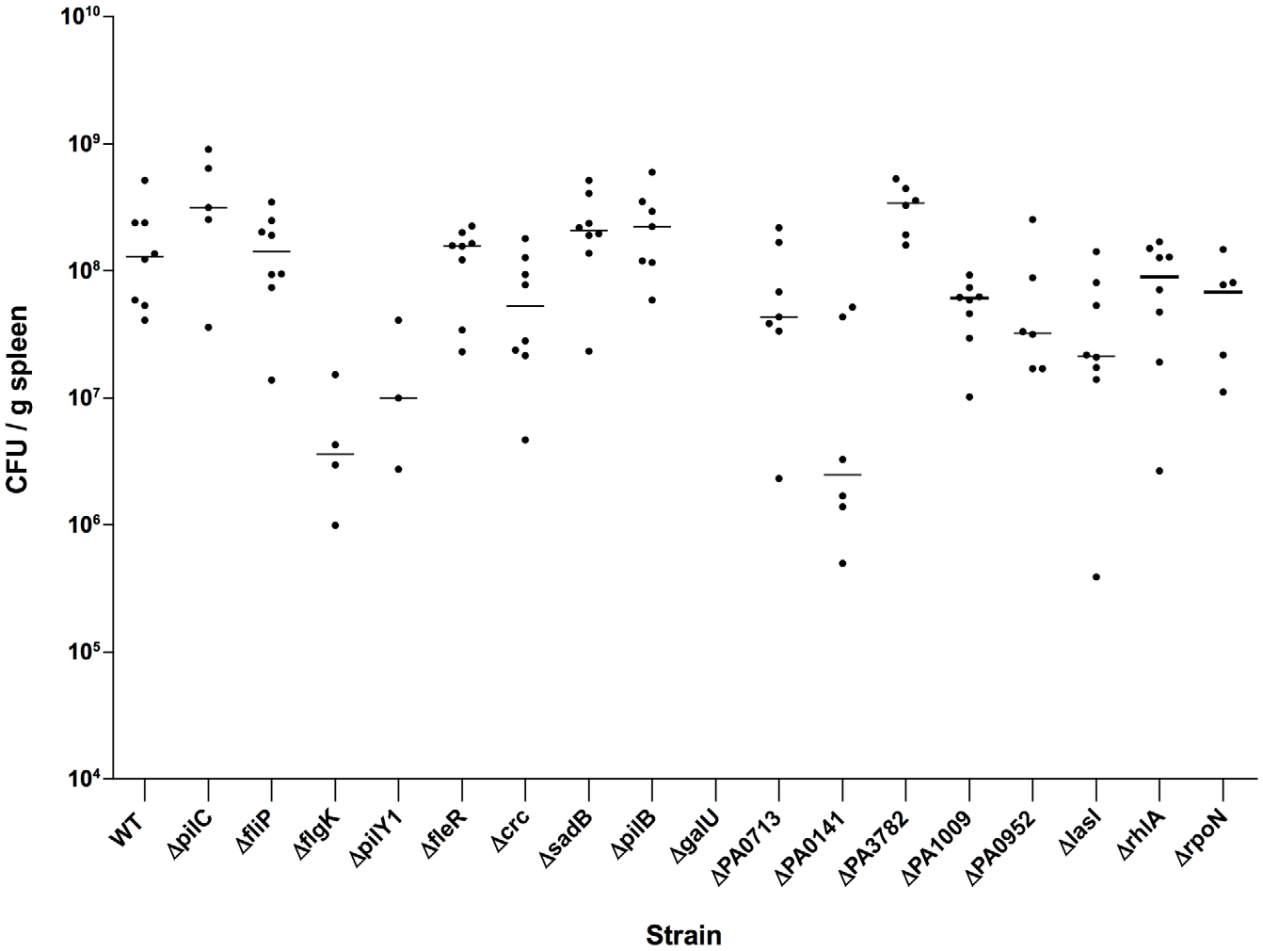
Viable counts of *P. aeruginosa* strains PA14 and PA14 Tn-mutants recovered from murine spleens. Points represent result from individual animals (female 6–8 wk-old C3H/HeN mice), and horizontal lines represent the medians. n = 6–8 for each strain (please refer to Table 3 for actual numbers for each mutant strain). Mice were initially colonized with PA14 and then subsequently made neutropenic with monoclonal antibody RB6-8C5 (0.200 mg IP once). Tn, transposon-insertion.

To verify *P. aeruginosa* bacteremia and dissemination had occurred in the moribund/dead animals, spleens from these mice were resected and homogenized. Homogenates were serially diluted and plated on both TSA and cetrimide agar. The presence of green, oxidase-positive colonies on cetrimide agar and absence of any heterogeneous colony types on TSA agar confirmed the presence of *P. aeruginosa*. All deceased mice had only *P. aeruginosa* recovered from the spleens, ranging in levels from 10^6^ to 10^9^ CFU/g spleen (Figure 5). As with the Tn-mutant strains recovered from the feces, maintenance of the mutant genotype following dissemination was also confirmed with colony PCR performed on *P. aeruginosa* grown from spleen homogenates.

### Colonization and Dissemination of *P. aeruginosa* Type III Secretion Mutants in the murine GI tract

Given that the uniform high expression levels of the genes involved in T3SS detected during GI colonization, we investigated the colonization and dissemination phenotypes of *P. aeruginosa* strains wherein the *exoS*, *exoT* or *popB* genes were deleted in strains PAO1 and PAK as well as the transposon insertional mutants in T3SS genes including *exoU* in the PA14 background. All three wild-type strains colonized the mouse GI tract at levels comparable to those previously reported [7]: *P. aeruginosa* strain PAK (n = 8, median = 1.35×10^8^ cfu/g stool, first quartile = 1.03×10^8^, third quartile = 1.93×10^8^); strain PA14 (n = 8, median = 5.60×10^7^ cfu/g stool, first quartile = 2.85×10^7^, third quartile = 8.77×10^7^); and strain PAO1 (n = 8, median = 1.19×10^8^ cfu/g stool, first quartile = 7.21×10^7^, third quartile = 1.35×10^8^). All T3SS mutant strains colonized the murine GI tract at levels comparable to or even higher (in the PA14 mutants) than their respective background strains (data not shown). Thus, the T3SS does not appear to be critical for GI colonization.

**Figure 6:**
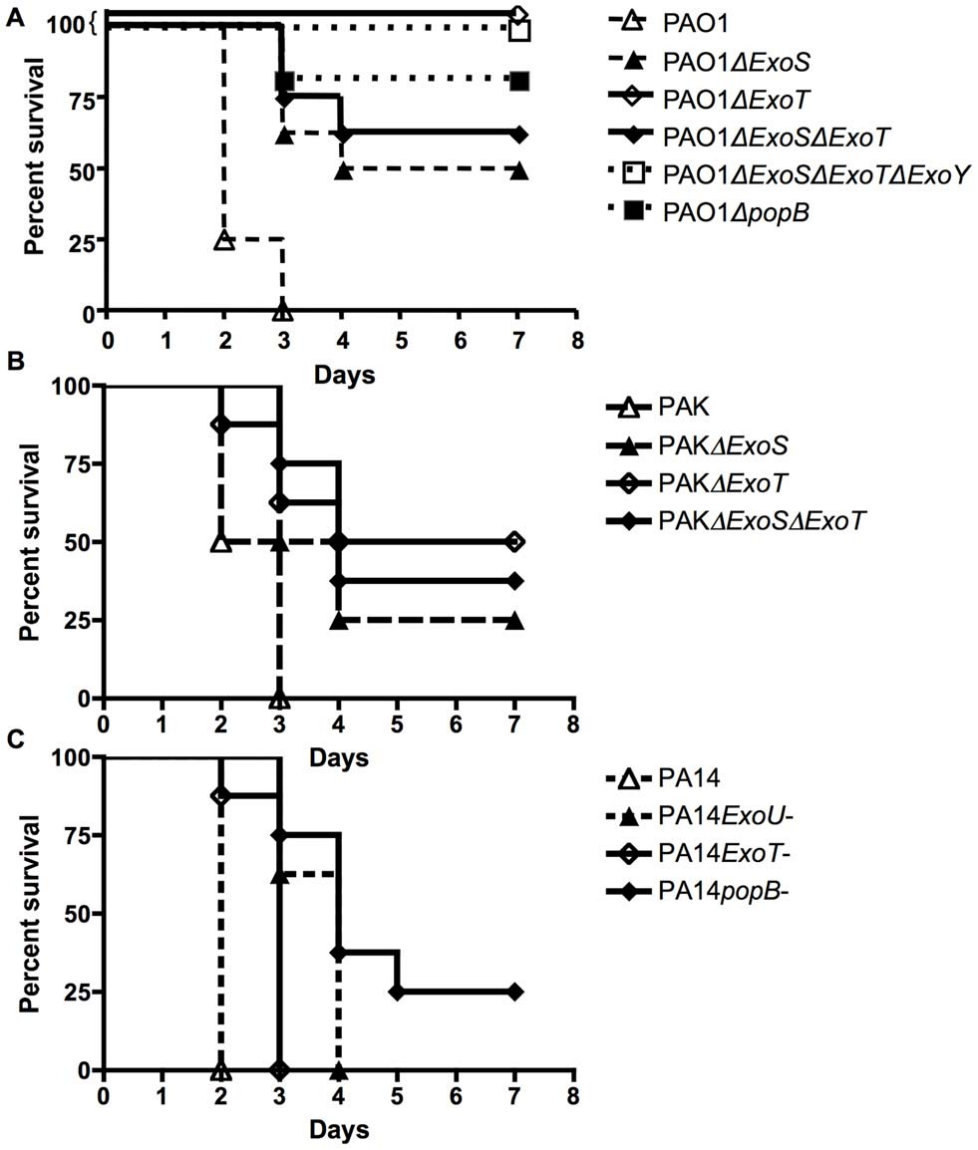
Survival curves of neutropenic mice colonized with wild-type *P. aeruginosa* and T3SS mutant strains. Female 6–8 wk-old C3H/HeN mice were colonized with *P. aeruginosa* strains PAO1(A), PAK (B), and PA14 (C) and with various deletional or insertional mutations of the type III secretion system in the corresponding WT background and then subsequently made neutropenic with monocloncal antibody RB6-8C5 (0.200 mg IP once). Median survival of mice colonized with mutants was significantly higher than that of mice colonized with the corresponding wildtype strain (P<0.009 for PAO1 mutants, P<0.009 for PAK mutants, P<0.006 for PA14 mutants, log-rank test). Each group contained 8 mice. T3SS, type III secretion system.

After the mice were made neutropenic by administering mAb RB6-8C5, 100% of mice colonized with WT strains PAK (n = 8), PA14 (n = 8), or PAO1 (n = 8) became moribund and were euthanized or died between observation periods. In contrast, there were significant differences in the length of survival between the WT and all their isogenic T3SS mutant strains (Figure 6) indicating that dissemination of *P. aeruginosa* in the setting of neutropenia is modified by the T3SS. However, in these experiments an overall mouse survival of ≤37.5% is not statistically different from the 100% lack of survival following dissemination of the WT parental strains, and while this was observed for all of the T3SS mutants in strain PAO1, only the mice infected with PAKΔ*exoT* strain had an overall significantly higher survival in this strain background. While mice infected with the PAK double Δ*exoS*Δ*exoT* mutant did not survive any better than the mice infected with the parental PAK strain, the difference in survival between mice infected with the single Δ*exoT* mutant and double Δ*exoS*ΔexoT strains was only 1 animal, likely indicative of normal experimental variation. None of the mice infected with the PA14 T3SS-deficient strains had significantly greater overall survival compared to survival of mice infected with the parental PA14 strain.

## Discussion

*P. aeruginosa's* pathogenic signature revolves around its ability to cause infectious in many clinical settings: ventilator-associated pneumonia, chronic lung infections in CF patients, skin and soft tissue infections of burn victims, and bacteremia and sepsis in cancer patients. The pathogenesis of *P. aeruginosa* bacteremia in cancer patients derives from a combination of a chronic, usually non-pathogenic state of GI colonization followed by an acute, invasive infection caused by extra-intestinal bacterial translocation and subsequent bacteremia once the immune system is compromised [9], [10], [27]. Using a murine model of *P. aeruginosa* GI colonization and neutropenia-induced dissemination, we have shown that factors critical for establishing disseminated disease in this model encompass the ability to establish GI colonization at quantitatively sufficient levels (e.g. a minimum of 10^7^ cfu/g stool) to allow for dissemination from the GI tract once neutropenia is induced [7]. We hypothesized that a microarray analysis of *in vivo* expressed mRNA transcripts during GI colonization would identify genes and/or phenotypes predictive of essentiality for the initiation and/or maintenance of the colonization state. However, while increased transcription of genes involved in both biofilm formation and T3SS were found during GI colonization, mutations in genes needed for these phenotypes did not alter the ability of *P. aeruginosa* to colonize the murine GI tract. We did find that loss of T3SS factors significantly increased the length of survival of mice infected with these mutants, but there was a variable effect of loss of the T3SS on overall mouse lethality. In general, our results indicate that increased mRNA transcription during *in vivo* infection may not be indicative of the need for the encoded protein in the infectious process.

We chose to use the PAK strain for these transcription profiling experiments because we had the greatest experience using this particular *P. aeruginosa* strain for microarray analysis[18], and at the time of these initial experiments, the PA14 TN library was not available. We only decided to utilize the PA14 TN mutants subsequent to the initial transcription profiling experiments. However, in retrospect the use of a different *P. aeruginosa* strain background to validate the results with specific Tn mutants based on transcriptome-findings with a heterologous strain probably adds robustness to the data as strain-specific effects are less likely to have been analyzed in the follow-up studies with the Tn mutants.

In addition to genes encoding proteins needed for biofilm formation and T3SS, 40% of genes involved in Cell Wall/LPS/Capsule production were generally increased by two-fold or more over expression when the bacteria are in water. The ability of *P. aeruginosa* to synthesize a complete LPS outer core [24] appears to be critical for establishing GI colonization in this murine model [7]. LPS and O-antigen synthesis have also been shown to be critical in establishing GI colonization with *Yersinia pseudotuberculosis* [28], *Yersinia enterocolitica* [29], and *E. coli* [30]. Thus, we did not explore these phenotypes in more depth as the need for production of an intact LPS by *P. aeruginosa* to establish and maintain GI colonization has been validated.

Son et al. [6] reported on *P. aeruginosa* transcript expression in CF sputum and found a limited number of transcripts expressed *in vivo* compared to those expressed by the same *P. aeruginosa* strain grown *in vitro*. This was surprising given the observation of specific phenotypes of the organisms found *in vivo*, such as elaboration of alginate, not correlating with increased transcription of synthetic or regulatory genes known to affect this phenotype. Further analysis indicated that the clinical isolate had increased constitutive expression of mRNA transcripts when grown *in vitro* when compared to the mRNA expression in the sequenced PA01 strain, indicating that many of the phenotypes that had emerged during chronic infection were based on genetic changes that were not markedly affected by transfer to the *in vitro* state for bacterial growth. When these findings are factored into our results, it seems that a quantitative increases *in vivo* expression of *P. aeruginosa* mRNA transcripts over that of a comparator *in vitro* state may have a limited role in predicting genes involved in the overall pathogenesis process.

In our experimental data set we found that 90% of the genes involved in T3SS had increased expression levels compared with *P. aeruginosa* in water, and in fact, almost 50% of these genes had expression levels >10-fold compared to baseline (Table 1) and none had decreased expression compared to baseline levels. However, this increased transcription of the T3SS genes did not predict a role in GI colonization but was associated with increases in the time of mouse survival following dissemination after induction of neutropenia. This suggests an importance of these effectors in the pathogenic process directed to bacterial translocation out of the GI tract and the associated ability of the T3SS effectors to systemically intoxicate host cells and tissues. Of note, there was not a uniform effect on overall survival in all three *P. aeruginosa* strains resulting from a loss of T3SS. With strain PAO1 all of the groups of mice infected with T3SS mutants had increased survival, whereas with strain PAK the group of mice infected with the Δ*exoT* mutant, but not the mice infected with the Δ*exoS* or double Δ*exoS/*Δ*exoT*, survived better than the parental group. With strain PA14 none of the mice infected with the T3SS mutants had better overall survival than mice infected with the parental strain. In the setting of experimental lung infection in animals evaluating the ExoU cytotoxin [31] as well as data from human *P. aeruginosa* lung infections [32], [33], [34] there is an indication of a role of the T3SS in virulence, although deletion of individual T3SS effectors in strain PAO1 had little effect on overall bacterial replication and survival in the lung [35], and only *popB*- and *exoSTY*-deleted mutants had a defect in systemic spread from the lung. All of these findings, including those reported here, not only emphasize the effect of strain background on the properties of mutant phenotypes but also raise questions as to the degree to which the T3SS is a factor in *P. aeruginosa* virulence among clinical isolates and in which settings of infection this virulence factor is essential.

In work previously reported by this laboratory, we demonstrated using immunohistochemical examination of intestinal tissues (cecal) obtained from mice colonized with *P. aeruginosa* that clumps of bacteria were trapped within the mucus layer of the murine cecums, while few bacterial cells were associated with the underlying epithelial cells[36]. Thus it appears that *P. aeruginosa* is embedded in an extracellular matrix *in vivo*, reminiscent of the phenotype commonly referred to as a biofilm mode of growth [37]. Consistent with these observations are results from histochemical staining and electron microscopy of lung tissue specimens obtained at autopsy from cystic fibrosis patients that also showed that aggregates of *P. aeruginosa* cells are seen *in vivo*, and, like the cells seen in the murine GI tract, are associated with the mucus and sequestered away from the epithelial surfaces of the airways [38]. Therefore, there appears to be direct evidence indicating that *P. aeruginosa* forms biofilms in both the GI and respiratory tracts within the mucus layer, but our results indicate that genes identified as being needed for *P. aeruginosa* biofilm formation *in vitro* are not needed for the mode of growth of the aggregated bacterial cells observed *in vivo*.

To fully test whether the biofilm-related genes that had increased expression *in vivo* GI colonization were strong candidates for further evaluation, we compared our microarray results with those from published studies analyzing the *in vitro* transcriptomes of *P. aeruginosa* cells growing in a biofilm to try and obtain a comprehensive identification of genes with increased transcription during biofilm growth. This was predicated on the lack of a consensus from multiple *in vitro* studies identifying a clear set of factors contributing to the biofilm phenotype, as this phenotype itself can be generated by different *in vitro* conditions. When comparing the GI colonization transcriptome data with previously published studies of *in vitro P. aeruginosa* biofilm formation, we ended up choosing twelve *P. aeruginosa* strains with interruptions in genes related to biofilm production for full *in vivo* analysis (*crc, fleR, flgK, fliP, PA3782, pilB, pilC, pilY1, sadB, lasI, rhlA*, and *rpoN)* even though only 6 of these genes were found to have mRNA transcripts increased by two-fold or more in vivo: *flgK, PA3782, pilB, pilC, pilY1,* and *sadB.* Of note, all of the genes encoding hypothetical proteins associated with *in vitro* biofilm production showed expression levels that were increased by two-fold or more in vivo, ranging from 4.4 to 24.8 fold increases from baseline (Table 2). Nonetheless, even by expanding our *in vivo* analysis to include evaluation of the need for genes found to be involved in *in vitro* biofilm formation that did not have increased expression *in vivo*, we still could not validate a role for biofilm formation in *P. aeruginosa* colonization of the GI tract and dissemination following induction of neutropenia.

Most of the biofilm-defective strains chosen for *in vivo* analysis showed this phenotype *in vitro*. We chose to utilize two different types of plastic to detect biofilm formation, PS and PP, since prior investigators had shown that some mutations resulted in loss of biofilm formation only on specific substrates, [20]. We found that with the exception of PA14Δ*rhlA*, which formed WT-levels of biofilms on both PS and PP, all the remaining mutant strains were attenuated in biofilm formation on both substrates (Fig. 2). Even after correcting for growth differences, the majority of mutant strains continued to show attenuated biofilm formation; the exceptions that showed WT biofilm formation with the correction for slower growth were the *rhlA* mutant on PS and the *pilC* mutant on PP (Fig. 3). What was most striking, however, was that the *galU* strain that we include as a negative control for murine GI colonization had a significantly increased ability to form biofilms *in vitro*, with a growth rate comparable to WT *P. aeruginosa* PA14 (Fig. 3). However, one of the limitations of the biofilm microtiter assay is that it is an endpoint assay and provides no information about biofilm developmental stages such as those that can be analyzed in flow cells. Nonetheless, we tested mutants with interruptions in both the *sadB* [19] and *lasI* [39] genes that have been shown to affect biofilm formation in dynamic flow cell assays and found they were also not attenuated in our *in vivo* system. While it is not clear which *in vitro* assay, if any, is useful for identifying strains attenuated for biofilm formation *in vivo*, which in and of itself is not a defined entity, our results suggest that the most commonly used *in vitro* methods do not reliably identify genes needed for *in vivo* virulence.

Only one *P. aeruginosa* PA14 mutant, the Δ*crc* strain exhibited a defect in GI colonization, which was nearly ten-fold lower than the wild-type strain (Fig. 4). While it is unclear if differences in colonization levels are physiologically significant, especially since the *crc* mutant caused comparable mortality to that of the WT PA14 following induction of neutropenia, it is conceivable that in a murine host colonized with *P. aeruginosa* along with other indigenous microbial cells, including anaerobic species, that a one log decrease in colonization rate may prevent disseminated disease from developing. We have found that a certain threshold level of *P. aeruginosa* GI colonization (e.g. ∼5×10^7^ cfu/g stool) is needed in order for translocation to occur once neutropenia is induced (AYK and GPB, unpublished observation).

In conclusion, we have utilized several independent molecular and genetic approaches to investigate whether *P. aeruginosa* genes noted to be important in *in vitro* biofilm formation exhibited any specific phenotype in a murine model of GI colonization and neutropenia-induced dissemination. Ultimately, we found that attenuation of biofilm formation in a static *in vitro* microtiter well did not correlate with a significant change in GI colonization level or mortality secondary to disseminated disease. These findings, however, do not completely diminish the importance of biofilm formation by *P. aeruginosa* with regards to GI tract colonization. Collections of microbial cells that could be called a biofilm have previously been described in the mucus covering the intestinal epithelial wall and on particulate matter in the intestine [40], [41]. It is possible that *P. aeruginosa* does not need any of its own gene products to make a biofilm *in vivo*, as the mucus layer of the intestine is believed to entrap microorganisms [42] and thus may provide the environment needed for *P. aeruginosa* cells to grow close together. If the microorganism can also survive the antimicrobial factors excreted by the host [43] and can proliferate faster than the mucus is being shed [44], then a microbial reservoir is created [45]. The mucus layer could potentially act as an entrapping matrix, similar to the exopolysaccharide (EPS) matrix secreted by biofilm forming organisms [46]. Thus, while all 16 genes in *P. aeruginosa* that were thought to be needed for biofilm formation *in vitro* had no appreciable phenotype *in vivo,* indicating that targeting these genes for disruption by therapeutic interventions likely will have little effect of the pathogenesis on infection and disease, there may still be potential interventions in the mucosal colonization process that could reduce the ability of *P. aeruginosa* to cause serious systemic infections. Similarly, we found that while transcription levels of T3SS genes were markedly elevated in the colonization state versus the basal state, the phenotype observed was not of decreased GI colonization but of decreased dissemination and yet there was also a variable effect from loss of the T3SS when measuring overall survival of neutropenic mice infected with mutant strains. Ultimately microarray analysis of *in vivo* transcriptomes to define genes needed for virulence may not be a particularly accurate tool and it seems likely better methods and experimental designs will be needed to obtain a more accurate prediction of virulence factors that impact *P. aeruginosa* virulence.

## Materials and Methods

### Bacterial strains and growth

The strains of *P. aeruginosa* used are listed in Table 4. The ordered transposon library in strain PA14 [13] was kindly provided by Dr. Fred Ausubel, Boston, MA. *P. aeruginosa* strains were grown overnight at 37°C in Luria Bertani (LB) broth (with the addition of 0.015 mg gentamicin/mL for PA14 transposon-insertion mutants), harvested by centrifugation, washed with PBS, and resuspended in PBS in preparation for *in vitro* biofilm assays as well as for inoculation into the drinking water to establish GI colonization in mice. The *P. aeruginosa* concentration was estimated using a spectrophotometer and actual cfu counts verified by enumeration on LB and cetrimide agars.

**Table 4:** Bacterial strains and genotype used in this study.

Strain	Relevant genotype or description	Source or reference
PAO1	Wildtype, serotype O2/O5, noncytotoxic, chloramphenicol sensitive, pilC^+^	M. Vasil
PAO1Δ*exoS*	PAO1Δ*exoS*	[35]
PAO1Δ*exoT*	PAO1Δ*exoT*	[35]
PAO1Δ2TOX	PAO1Δ*exoS*Δ*exoT*	[35]
PAO1Δ3TOX	PAO1Δ*exoS*Δ*exoT*Δ*exoY*	[35]
PAO1Δ*popB*	PAO1Δ*popB*	[35]
PAK	Wildtype	S. Lory
PAKΔ*exoS*	PAKΔ*exoS*	S. Lory
PAKΔ*exoT*	PAKΔ*exoT*	S. Lory
PAKΔ2TOX	PAKΔ*exoS*Δ*exoT*	S. Lory
PA14	Wildtype, serogroup O10 strain, cytotoxic (ExoU^+^)	[50]
PA14*crc-*	*crc*::MAR2xT7^a^ (Gm^R^, 15 µg/ml)	[13]
PA14*fleR-*	*fleR*::MAR2xT7 (Gm^R^, 15 µg/ml)	[13]
PA14*flgK-*	*flgK*::MAR2xT7 (Gm^R^, 15 µg/ml)	[13]
PA14*fliP-*	*fliP*::MAR2xT7 (Gm^R^, 15 µg/ml)	[13]
PA14*galU-*	*galU*::MAR2xT7 (Gm^R^, 15 µg/ml)	[13]
PA14*pilB-*	*pilB*::MAR2xT7 (Gm^R^, 15 µg/ml)	[13]
PA14*pilC-*	*pilC*::MAR2xT7 (Gm^R^, 15 µg/ml)	[13]
PA14*pilY1-*	*pilY1*::MAR2xT7 (Gm^R^, 15 µg/ml)	[13]
PA14*sadB-*	*sadB*::MAR2xT7 (Gm^R^, 15 µg/ml)	[13]
PA14*PA3782-*	*PA3782*::MAR2xT7 (Gm^R^, 15 µg/ml)	[13]
PA14*PA0141-*	*PA0141*::MAR2xT7 (Gm^R^, 15 µg/ml)	[13]
PA14*PA0713-*	*PA0713*::MAR2xT7 (Gm^R^, 15 µg/ml)	[13]
PA14*PA0952-*	*PA0952*::MAR2xT7 (Gm^R^, 15 µg/ml)	[13]
PA14*PA1009-*	*PA1009*::MAR2xT7 (Gm^R^, 15 µg/ml)	[13]
PA14*lasI-*	*lasI*::MAR2xT7 (Gm^R^, 15 µg/ml)	[13]
PA14*rhlA-*	*rhlA*::MAR2xT7 (Gm^R^, 15 µg/ml)	[13]
PA14*rpoN-*	*rpoN*::MAR2xT7 (gm^R^, 15 µg/ml)	[13]
PA14*exoU-*	*exoU*::MAR2xT7 (gm^R^, 15 µg/ml)	[13]
PA14*exoT-*	*exoT*::MAR2xT7 (gm^R^, 15 µg/ml)	[13]
PA14*popB-*	*popB*::MAR2xT7 (gm^R^, 15 µg/ml)	[13]

a MAR2xT7 is a Himar1-derivative that originates from the eukaryotic mariner transposon-family [13].

Gm^R^: Gentamicin resistance.

### Production or RB6-8C5 monoclonal antibody and induction of neutropenia

The RB6-8C5 mAb specific for the Ly6 antigen that is highly expressed by polymorphonuclear neutrophils (PMN) was produced by growth of hybridoma cells in culture (Dulbecco's modified Eagle's medium with 10% fetal calf serum (FCS)) followed by purification of antibody by protein G chromatography, as previously described [7]. A single dose of 200 µg of RB6-8C5 was administered to C3H/HeN mice to produce a severe neutropenia (absolute neutrophil count <100) for 5 days [7].

### Murine model of P. aeruginosa GI colonization and neutropenia-induced dissemination

We used a murine model of *P. aeruginosa* GI colonization and neutropenia-induced dissemination as previously described [7] with minor modifications. Six- to 8-week-old female C3H/HeN mice (Harlan Laboratories) were housed as groups of 4 in sterilized cages equipped with filter hoods. Mice were supplied with sterile bedding, sterile water and sterile mouse chow and maintained under specific pathogen-free conditions at the animal facilities of the Harvard Medical School in compliance with the Harvard Medical Area Institutional Animal Care and Use Committee guidelines and at the animal facilities of the University of Texas Southwestern Medical Center in compliance with the Institutional Animal Care and Use Committees of the University of Texas Southwestern Medical Center. To deplete the indigenous GI bacterial flora, mice were fed sterile water with 2 mg streptomycin/mL (Research Product International, Mt. Prospect, IL) and 1500 U penicillin G/mL (Sigma-Aldrich, St. Louis, MO) for 4 days. Stool was collected from individual mice (0.030–0.050 g per stool pellet), homogenized in 1 mL 1% proteose peptone, and 100 µl of the homogenate was spread on trypticase soy (TSA) and MacConkey agars and grown overnight at 37°C to verify reduction of indigenous GI microbial flora. *P. aeruginosa* strains were grown as described above, added to sterile water with 1500 Units penicillin G/mL at approximately 10^7^ cfu/ml, and then administered to mice for 5 days. Bacterial water was changed every second day to maintain adequate cfu levels. After 5 days of exposure to *P. aeruginosa*, stool was again collected, homogenized in 1 mL 1% protease peptone, serially diluted in 1% proteose peptone and dilutions plated on TSA and LB agar (with 0.015 mg gentamicin/mL (Research Product International, Mt. Prospect, IL) for PA14 transposon insertion mutants) to measure levels of GI colonization by *P. aeruginosa*. Once colonization with *P. aeruginosa* was confirmed, 200 µg of mAb RB6-8C5 was administered to each mouse by IP injection. For mice colonized with PA14 transposon insertion mutants, sterile water with 1500 Units penicillin G/mL and 0.015 mg gentamicin/mL was initiated after RB6-8C5 administration. Mice were monitored for morbidity for 7 days. Moribund mice were euthanized and along with mice found dead between observation periods, the carcasses were frozen at minus 20°C, later thawed, spleens were resected, homogenized in 1 mL 1% protease peptone, serially diluted and 10 µl of the homogenate was drip-plated on TSA and LB (with or without 0.015 mg gentamicin/mL) agar plates. Growth media were incubated at 37°C overnight under aerobic conditions. The presence of green oxidase-positive colonies was used for confirmation of *P. aeruginosa* systemic dissemination.

### Ethics Statement

This study was carried out in strict accordance with the recommendations in the Guide for the Care and Use of Laboratory Animals of the National Institutes of Health. The protocol was approved by the Harvard Medical Area Institutional Animal Care and Use Committee (Permit Number: 404-R98) and the Institutional Animal Care and Use Committees of the University of Texas Southwestern Medical Center (Permit Number: 2009-0243). All efforts were made to minimize suffering.

### RNA Isolation

In order to establish a comparative transcriptome for *P. aeruginosa* isolated from colonized mice, we isolated *P. aeruginosa* RNA from bacteria in the drinking water provided to the mice. Sterile water with 1500 Units penicillin G/mL and *P. aeruginosa* strain PAK (10^7^ cfu/ml) was stored in a sterile flask for 24 hours. Bacteria were harvested by centrifugation, and the RNA isolation protocol described below was initiated.

To obtain transcriptome information from bacteria colonizing the murine GI tract, we initiated the murine model utilizing the *P. aeruginosa* PAK strain. Seven days after initiating *P. aeruginosa* drinking water and two days after confirmation of GI colonization with PAK, eight mice were euthanized and the cecal contents were flushed with a buffer containing 10 mM TrisHCl, 1 mM EDTA and 200 mM NaCl [47] into a sterile stainless steel mortar (Fisher Scientific, Pittsburgh, PA) which was immediately immersed in liquid nitrogen. Cecal flushate contents were then ground with a sterile pestle on a stainless steel mortar immersed in liquid nitrogen, then added to 0.5 volume of boiling lysis buffer (2% SDS, 16 mM EDTA, 200 mM NaCl), homogenized, and kept at 100°C for 5 minutes with frequent mixing. A hot acid phenol:chloroform(5∶1 ratio of phenol:chloroform, Ambion, Foster City, CA) extraction was carried out at 65°C. An additional 2–3 room temperature acid phenol:chloroform extractions were performed [48] followed by a final chloroform:isoamyl alcohol extraction. Isopropanol (Sigma-Aldrich, St. Louis, MO) precipitation at −20°C, overnight was followed by treatment with RQ1 DNase (Promega Corporation Madison, WI) for 1 hour at 37°C to remove contaminating DNA. After DNase treatment and additional cold acid phenol:chloroform extractions, a final chloroform:isoamyl alcohol extraction was performed. The aqueous fraction was then precipitated with 2 volumes of 100% ethanol and 0.1 volume of 3 M sodium acetate (pH 5.5) at −80°C for 30 minutes. RNA was further purified with RNeasy Mini RNA purification kit (RNA cleanup protocol; Qiagen, Valencia, CA). Samples were eluted in 60 ul of RNase-free water. Purity was confirmed by agarose gel electrophoresis and spectrophotometry (Nanodrop Technologies, Wilmington, DE). No evidence of eukaryotic rRNA (18 s at 1.9 kb and 28 s at 5 kb) was detected using Northern analysis of ethidium bromide stained gels. All RNA samples used for cDNA synthesis had 260/280 ratios ranging from 1.7 to 2.0.

### cDNA Synthesis and Microarray Hybridization

cDNA was synthesized and hybridized to micoarray chips as previously described [18]. The protocol and modifications were as follows: 12 micrograms of purified total RNA extracted from pooled murine cecal samples (8 mice) or the drinking water containing strain PAK were converted to cDNA with Superscript II Reverse transcriptase (Invitrogen, Carlsbad, CA) and a semi-random decamer, termed (NS)_5_ (Invitrogen Life Technologies, custom oligonucleotide order, 5′NSNSNSNSNS 3′, where N = A,T,C, or G and S = C or G). As a control for cDNA production and microarray hybridization, control RNAs were spiked into the reverse transcriptase reaction. The resulting cDNA was purified, partially digested with DNase I, and end labeled with ddUTP-biotin (Enzo Life Sciences, Farmingdale, NY). The resulting targets were hybridized to GeneChip *P. aeruginosa* Genome Arrays (Affymetrix,). Labeled DNA was hybridized to Affymetrix *P. aeruginosa* Genome Arrays, and chips were washed, stained and scanned as previously described [18].

### Microarray Analysis and Statistical Significance Assessment

Hybridization intensity data was collected from the scanned array images of three replicate experiments (for both baseline and GI colonization transcriptome levels), and intrachip normalizations were performed with Affymetrix Microarray Suite 5.0 software. Statistical analysis and experimental comparisons were made with GeneSpring version 4.2.1 (Agilent Technologies, Santa Clara, CA). Significance was established on the basis of three criteria: first, a Welch t-test was performed to determine whether the difference in expression for a given gene was greater between test conditions than within replicates of the same condition. For this test, a probability cutoff of 0.1 was used (p<0.1). Next, the average hybridization intensity for each probe set had to be above background in the activating condition. This determination was made on the basis of the default absolute call metric in Microarray Suite 5.0 (Affymetrix). Third, the magnitude of change between two conditions (baseline drinking water and GI colonization state) had to be greater than 2-fold [18]. Data generated from three separate microarrays were used to calculate average transcription levels. All microarray data is MIAME compliant and has been deposited into the Gene Expression Omnibus database (accession number GSE22665).

### Colony PCR and gel electrophoresis

A single colony was selected from a plate, transferred into 30 µl ddH_2_O and boiled at 100°C for 5 min. Cell debris was pelleted by centrifugation (2 min at 16,000×g), and supernatants transferred into a new tube and stored on ice. The supernatant was used as DNA template for the PCR reaction, which contained 43 µl of the PCR Platinum Supermix (Invitrogen, Carlsbad, CA), 1 µl of each forward and reverse primer (10 µM) and 5 µl of DNA template combined in a PCR tube. Forward and reverse primers are listed in Table S1. PCR conditions were as follows: 94°C for 30 sec; 30 cycles of 94°C for 30 sec, 55°C for 30 sec and 72°C for 1 min.; final ext. at 72°C for 5 min. For gel electrophoresis, 1% agarose (American Bioanalytical, USA) in TAE gel was used. Samples were run at 100 V for 80 min and examined in a BioRad Gel Doc XR system (Biorad Laboratories, Hercules, CA).

All 20 of the mutants used that were retrieved from the *P. aeruginosa* PA14 transposon library (24) were confirmed to have the correct insert based on the results from the colony PCR. The *crc* mutant produced a blue/green phenotype when growing on LB-agar, similar to what was observed by others [22], [49].

### Microtiter plate biofilm assay

The *in vitro* biofilm assay was performed as previously described [20] with the following modifications. A *P. aeruginosa* Tn-mutant strain of interest was grown overnight at 37°C on LB agar plates, then suspended in sterile water with 1500 U Penicillin G/mL and 0.015 mg gentamicin/mL to a concentration of 10^9^ CFU/ml. No antibiotics were added for wild-type strains. *P. aeruginosa* bacterial suspensions were serially diluted to a final concentration of 1∶100; 100 µl was transferred to seven replicate wells using sterile PS 96-well plates (Costar 3596, Corning, Corning, NY) or PP 96-well plates (Costar 3364, Corning). Four wells in each plate were not inoculated (LB medium only) for negative control references. Plates were sealed with parafilm and incubated for 12–14 hours at 37°C. Growth was quantified by using a 96-well ELISA reader and a wavelength of 630 nm (OD_630_) (ELx800, Bio-tek Instruments, Winooski, VT). Microtiter plate wells were emptied and washed twice in 130 µl 0.9% sterile NaCl. 200 µl 1% crystal violet was added to each well and incubated for 15 min at RT. Wells were then emptied, washed twice in 130 µl sterile 0.9% NaCl and destained with 200 µl 95% ethanol for 5 min. Biofilm formation was quantified using a 96-well ELISA Plate reader and an OD_595_ wavelength.

For *in vitro* biofilm formation under anaerobic conditions, we utilized the same protocol as described above with the following exceptions. *P. aeruginosa* strains were suspended in LB broth (with or without gentamicin) containing 15 mM KNO_3_ at pH 6.5 and grown anaerobically in a Coy anaerobic chamber at 37°C for 24 hours [3].

### Statistical Analyses

Survival data were analyzed by Fisher's exact test and log rank test, and two-way comparisons of GI colonization levels were carried out using the Kruskal-Wallis with Dunn's multiple comparison test to a single control group using the GraphPad Prism software (San Diego, CA). When multiple comparisons or more than two groups were analyzed, Bonferroni's correction to the significance level α was invoked.

## Supporting Information

Table S1 Primer Names and sequences.
(DOC)
