# Supplementary material for: Utility of In Vivo Transcription Profiling for Identifying Pseudomonas aeruginosa Genes Needed for Gastrointestinal Colonization and Dissemination
Source: PLoS One. 2010 Dec 10;5(12):e15131. doi: 10.1371/journal.pone.0015131 (PMC3000825; doi:10.1371/journal.pone.0015131)
Supplement: Table S1 — Primer Names and sequences. (DOC) [file pone.0015131.s001.doc]

Table S1. Primer names and sequences

| **Primer Name** | **Sequence 5’-3’** |
| --- | --- |
| *crc* F | GGGGACTTATCAGCCAACCT |
| *crc* R | GGTGGCGATACTCACCTTGT |
| *fleR* F | TAGAGACCGGGGACTTATCA |
| *fleR* R | CTCGTCGAGAAGAATGGTG |
| *flgK* F | TAGAGACCGGGGACTTATCA |
| *flgK* R | GAGTTGTTGAAAGCCTGGAT |
| *fliP* F | GGGACTTATCAGCCAACCT |
| *fliP* R | GAAGATCATGAAGCCGATCT |
| *galU* F | TAGAGACCGGGGACTTATCA |
| *galU* R | CTGCTCGATCAGATAGAAGATG |
| *pilB* F | TAGAGACCGGGGACTTATCA |
| *pilB* R | CACGAATGGTGTAATCCTGA |
| *pilC* F | GGGGACTTATCAGCCAACCT |
| *pilC* R | AAGGGTATGCCGTCACACAT |
| *phzB1* F | GGACTTATCAGCCAACCTGT |
| *phzB1* R | ACGAACTTCGCGAAAAGAAT |
| *sadB* F | TAGAGACCGGGGACTTATCA |
| *sadB* R | GTACAGGCAGGGCGAACT |
| *PA3782* F | TAGAGACCGGGGACTTATCA |
| *PA3782* R | GTGGCCTGAGAGACAACTTT |
| *PA0141* F | GGACTTATCAGCCAACCTGT |
| *PA0141* R | GTAGCGCTGGAAATACCACT |
| *PA0713* F | GGTCTAGAGACCGGGGACTT |
| *PA0713* R | TGGAAACGTTCATGTCGGTA |
| *PA0952* F | GGTCTAGAGACCGGGGACTT |
| *PA0952* R | CTCCCAATGTCGAACAAGGT |
| *PA1009* F | TAGAGACCGGGGACTTATCA |
| *PA1009* R | CGGGTAGAAATACAGGACGA |
| *lasI* F | CGTGCTCAAGTGTTCAAGGA |
| *lasI* R | GCGTCTGGATGTCGTTCTG |
| *rhlA* F | CCAGCAACCATCAGCACAT |
| *rhlA* R | GGCGGTGGTGTACTCGTC |
| *rpoN* F | AACGACGATGACGAATGG |
| *rpoN* R | CGCAGCACGACTTCCACTT |
| *exoT* F | GGAGACGTCAATCATCATGC |
| *exoT* R | CAGTTTGCCCAGCCACTC |
| *exoU* F | TGTGGCTGGCATTGAACACC |
| *exoU* R | TCGCTGAAAAAGGGCTTGG |
| *popB* F | AACCTGCAGAAGATGGAGGA |
| *popB* R | GAGACCACTCCCATGACACC |
